# Native myocardial longitudinal (*T*_1_) relaxation time: Regional, age, and sex associations in the healthy adult heart

**DOI:** 10.1002/jmri.25217

**Published:** 2016-03-04

**Authors:** Samuli M.O. Rauhalammi, Kenneth Mangion, Pauline Hall Barrientos, David J.A. Carrick, Guillaume Clerfond, John McClure, Christie McComb, Aleksandra Radjenovic, Colin Berry

**Affiliations:** ^1^ BHF Glasgow Cardiovascular Research CentreUniversity of GlasgowGlasgowUK; ^2^ West of Scotland Heart and Lung Centre, Golden Jubilee National HospitalGlasgowUK; ^3^ Department of Clinical PhysicsQueen Elizabeth University HospitalGlasgowGlasgowUK

**Keywords:** native *T*_1_, longitudinal relaxation time, *T*_1_ mapping, myocardium, healthy volunteer

## Abstract

**Purpose:**

To use magnetic resonance imaging (MRI) at two field strengths to assess healthy adults' regional myocardial noncontrast (native) *T*_1_ relaxation time distribution, and global myocardial native *T*_1_ between sexes and across age groups.

**Materials and Methods:**

In all, 84 healthy volunteers underwent MRI at 1.5T and 3.0T. *T*_1_ maps were acquired in three left ventricular short axis slices using an optimized modified Look–Locker inversion recovery investigational prototype sequence. *T*_1_ measurements in msec were calculated from 16 regions‐of‐interest, and a global *T*_1_ value from all evaluable segments per subject. Associations were assessed with a multivariate linear regression model.

**Results:**

In total, 1297 (96.5%) segments were evaluable at 1.5T and 1263 (94.0%) segments at 3.0T. Native *T*_1_ was higher in septal than lateral myocardium (1.5T: 956.3 ± 44.4 vs. 939.2 ± 54.2 msec; *P* < 0.001; 3.0T: 1158.2 ± 45.9 vs. 1148.9 ± 56.9 msec; *P* = 0.012). Native *T*_1_ decreased with increasing age in females but not in males. Among lowest age tertile (<33 years) global native *T*_1_ was higher in females than in males at 1.5T (960.0 ± 20.3 vs. 931.5 ± 22.2 msec, respectively; *P* = 0.003) and 3.0T (1166.5 ± 19.7 vs. 1130.2 ± 20.6 msec; *P* < 0.001). No sex differences were observed in upper age tertile (≥55 years) at 1.5T (937.7 ± 25.4 vs. 934.7 ± 22.3 msec; *P* = 0.762) or 3.0T (1153.0 ± 30.0 vs. 1132.3 ± 23.5 msec; *P* = 0.056). Association of global native *T*_1_ to age (*P* = 0.002) and sex (*P* < 0.001) was independent of field strength and body size.

**Conclusion:**

In healthy adults, native *T*_1_ values are highest in the ventricular septum. Global native *T*_1_ was inversely associated with age in women, but not in men. J. Magn. Reson. Imaging 2016;44:541–548.

Advances in magnetic resonance imaging (MRI) now enable the estimation of longitudinal (spin‐lattice, *T*_1_) proton relaxation time in vivo using parametric mapping techniques. Non‐contrast (native) *T*_1_ reflects myocardial water content and pathology, and *T*_1_ mapping has emerging clinical utility for detection of acute myocardial infarction,^1^ acute myocarditis,^2^ infiltrative cardiomyopathy,^3^, ^4^ and pressure‐overload hypertrophy.^5^

*T*_1_ can be measured in regions‐of‐interest (ROIs) in the heart.^6^ However, *T*_1_ map acquisitions are susceptible to artifacts, especially at higher magnetic fields, making their interpretation challenging.^7^ *T*_1_ values also vary between scanner type and pulse sequence, and clinical guidelines recommend standardization of image acquisition and analysis.^8^ Piechnik et al^9^ described variation of native myocardial *T*_1_ in healthy subjects using the shortened modified Look–Locker inversion recovery (ShMOLLI) method at 1.5T in subjects aged 11–69 years (mean ± standard deviation [SD] age 38 ± 15) years. They observed that native *T*_1_ was associated with sex, body size, and hematocrit, but not age. The current study is a further assessment of native *T*_1_ variation using a different *T*_1_ mapping method, at different MR field strengths, in older individuals, and involving gadolinium‐based contrast MR to rule out incidental myocardial disease.

## Materials and Methods

### Volunteers

Healthy adults across a broad age range were enrolled based on responses to advertisements on public noticeboards and through personal contacts of the investigators. All subjects gave written informed consent after the nature of procedures had been fully explained, and ethical approval was granted for all study procedures (West of Scotland Research Ethics Service, reference 11/AL/0190). The inclusion criteria were age >18 years, no known history of cardiovascular disease or systemic illness, and a normal 12‐lead electrocardiogram (ECG) recording. The exclusion criteria included prior history of cardiovascular or connective tissue disease, or treated hypertension or hypercholesterolemia. There was no upper age limit. For females, pregnancy or suspected pregnancy was also included as part of the exclusion criteria.

### MRI Protocol

MRI was performed at 1.5T (Magnetom Avanto, with a 12‐element phased array surface coil, Siemens Healthcare, Erlangen, Germany) in a large regional hospital and at 3.0T (Magnetom Verio, with a 16‐element phased array surface coil, Siemens Healthcare) in the university research center. The imaging protocol included cine MR with steady‐state free precession (SSFP) and *T*_1_‐relaxometry (mapping) sequences. A cine short axis (SA) stack covered the full left ventricle (LV). *T*_1_ maps were acquired in three SA slices (basal, mid, and apical), using a motion‐corrected optimized modified Look–Locker inversion recovery (MOLLI) investigational prototype sequence (Siemens Healthcare, works‐in‐progress method 448).^10^, ^11^ The MOLLI *T*_1_ cardiac‐gated acquisition involved three inversion‐recovery prepared inversion time (TI) scout experiments, with three heartbeats for recovery between each experiment, combined within one protocol (3 (3) 3 (3) 5).^12^ Typical imaging parameters are provided in Table 1.

**Table 1:** Typical Imaging Parameters at 1.5T and 3.0T

	1.5T	3.0T
Bandwidth	1090 Hz/pixel	930 Hz/pixel
Flip angle	35 °	35 °
Echo time (TE)	1.1 msec	1.06 msec
T1 of first experiment	100 msec	100 msec
TI increment	80 msec	80 msec
Repetition time (TR)	788 msec	740 msec
Parallel imaging	2	2
Partial Fourier	6/8	6/8
Matrix	192 × 124 pixels	192 × 124 pixels
Spatial resolution	2.2 × 1.8 × 8.0 mm	2.2 × 1.8 × 8.0 mm
Scan time	17 heartbeats	17 heartbeats

Participants over 45 years of age and an estimated glomerular filtration rate >30 mL/min underwent further contrast‐enhanced imaging. Delayed‐enhancement phase‐sensitive inversion‐recovery pulse sequences, covering the SA stack of a full LV,^13^ and three (basal, mid, and apical) postcontrast *T*_1_ maps were acquired 10–15 minutes after intravenous contrast agent administration at 1.5T. Contrast was 0.15 mmol/kg of gadolinium diethyltriaminepenta‐acetic acid (Magnevist, Bayer Healthcare, Berlin, Germany). Volunteers aged >45 years, who did not receive contrast, were included in the other analyses.

Two phantoms (small: cylindrical, diameter 15 cm; large: box‐shaped, 40 × 40 × 10 cm), containing water and contrast, were scanned at 1.5T and 3.0T. Phantoms were positioned centrally on the scanner table and axial *T*_1_ maps acquired with a simulated heart rate of 60 bpm.

### Image Analysis

Anonymized images were analyzed in a random order on a Siemens Healthcare (syngoMR) workstation by two MR‐trained observers (S.R., D.C.) with 4 years of cardiac MR experience. The accuracy of all of the image analyses was reviewed by a cardiologist with over 10 years of experience (C.B.) in cardiac MR. The overall image quality was ranked as high, adequate, or nondiagnostic, based on: endo‐ and epicardial border definition (ie, ECG gating), success of motion correction image alignment, presence and severity of ghosting (ie, breathing) and SSFP off‐resonance artifacts.

LV dimensions, volume, and ejection fraction were quantified using computer‐assisted planimetry and an axial stack of images, and compared against well‐established reference ranges.^14^ The late gadolinium enhancement (LGE) images, covering the entire LV, were evaluated visually following current clinical guidelines,^11^, ^15^ and the absence of myocardial LGE was a requirement for inclusion of the participant in the analysis.

Each *T*_1_ map was assessed separately by two observers (S.R., K.M.) for the presence of artifacts relating to susceptibility effects or cardiorespiratory motion, and evaluated against the original images. When there was discordance between the artifact scoring, a third observer (C.B.) acted as a blinded independent adjudicator. Artifacts related to off‐resonance in MOLLI SSFP readout were included in susceptibility artifacts. When artifacts occurred and observers unanimously agreed that these would potentially contribute to variation in the *T*_1_ (msec), the affected segments were not included in the analysis. LV contours were delineated on the raw *T*_1_ image and copied onto the color‐enhanced spatially coregistered maps. *T*_1_ maps were segmented according to the American Heart Association (AHA) 16‐segment model, using the anterior right ventricular‐LV insertion point as the reference point.^16^ Segmental AHA ROIs were delineated by user‐defined semiautomated border delineation (Argus, Siemens Healthcare). The ROIs were standardized to be of similar size and shape, containing at least 100 pixels in all of the segments. The *T*_1_ value was measured in each of the segments included, with particular care taken to delineate ROIs with adequate margins of separation from tissue interfaces prone to partial volume averaging, such as between blood‐pool and myocardium.^8^, ^15^ The ROI from LV blood pool was also measured. ROIs were copied between the pre‐ and postcontrast *T*_1_ maps. Typical *T*_1_ maps are shown in Supplementary Fig. 1.

**Figure 1:**
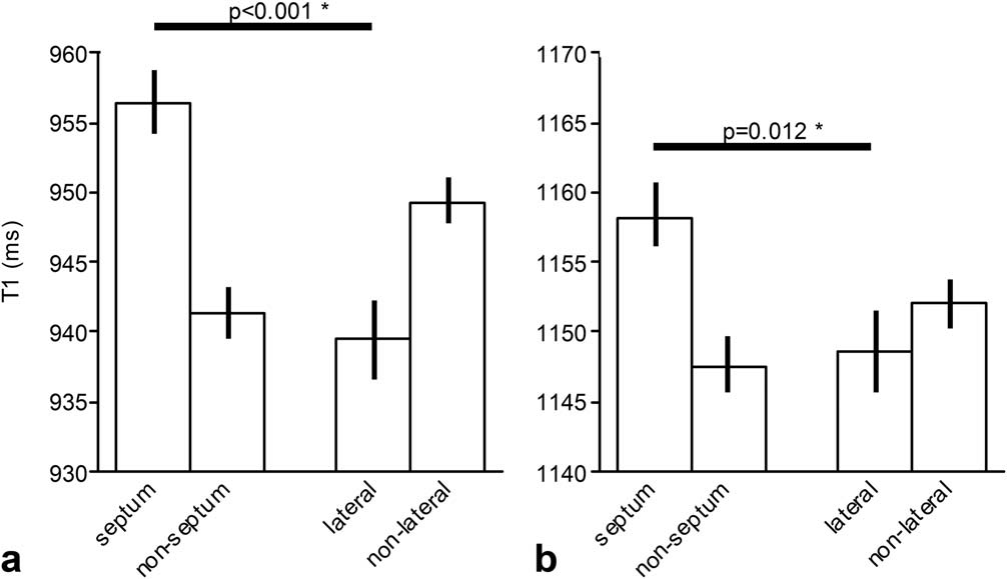
Regional differences in mean native *T*_1_ relaxation times (msec; mean, 95% CI) between septal vs. nonseptal ROIs and lateral vs. nonlateral ROIs, at: **a** = 1.5T and **b** = 3.0T.

Septal *T*_1_ was calculated as a mean value of anteroseptal, inferoseptal, and septal AHA segments, while nonseptal *T*_1_ is the mean of the remaining AHA segments. Lateral *T*_1_ refers to the mean of inferolateral, anterolateral, and lateral AHA segments, and nonlateral to the remaining segments. Global averaged myocardial *T*_1_ relaxation times are presented as a mean value of all analyzable segments on a per‐subject basis.

For the phantom analysis, ROIs containing at least 20 pixels were drawn to cover the entire area of the phantom *T*_1_ map. For the smaller phantom, 10 ROIs were used, and for the larger phantom 20 ROIs.

### Statistical Analysis

Categorical variables were expressed as number and percentage of observations. Normality was explored using residual plots and confirmed or excluded with the Ryan‐Joiner statistic. Continuous variables with normal distribution are presented as means ± SD unless otherwise mentioned. Extracellular volume (ECV) was calculated as ECV = (1‐HCT)*([1/*T*_1myo post_‐1/*T*_1myo pre_]/[1/*T*_1blood post_‐1/*T*_1blood pre_]).^17^ When a blood sample for hematocrit (HCT) was not available, an estimation HCT = 0.88‐(*T*_1blood_/3240) was used.^18^ Body surface area (BSA) was calculated using DuBois & DuBois method.^19^ Correlation analyses were Pearson tests. Regional, sex, and age differences were assessed by the unpaired *t*‐test, while comparisons between field strengths were undertaken with the paired *t*‐test. In order to assess for associations between anthropometry and *T*_1_, subjects were categorized by sex and age (tertiles with equal *n* values) and assessed using analysis of variance (ANOVA). No corrections were made for multiple testing. The univariate relationships between age, sex, height, weight, body mass index (BMI), and BSA were assessed, and univariate associates (*P* < 0.05) were then included in a multivariate linear regression analysis. For regression models, male sex was coded as 1 and female sex as 0. For all of the analyses *P* < 0.05 was considered statistically significant. Image analyst intra‐ and interobserver variability was tested in 30 volunteers selected at random per each field strength and assessed by Bland–Altman plots and 95% limits of agreement. The statistical analyses were performed using Minitab software (Minitab, State College, PA, v. 16.2.2).

## Results

In total, 86 healthy adults underwent MRI (1.5T and 3.0T) 1.4 ± 1.4 days apart. Two subjects did not complete the MRI protocol. One male had an incidental finding of high *T*_1_ in the anterior wall of the left ventricle in the distribution of the left anterior descending coronary artery and, when retrospectively reviewed, had exertional chest pain suggestive of angina, which was not disclosed previously. One female experienced claustrophobia. The characteristics of the participants with complete *T*_1_ MRI (*n* = 84) are shown in Table 2.

**Table 2:** Characteristics of the Healthy Volunteers

Overall (*n = * 84)	
Mean ± SD age, years	45 ± 18.0
Male sex, *n* (%)	43 (49.4)
Mean ± SD height, cm	171.1 ± 9.9
Mean ± SD weight, kg	77.1 ± 14.8
Mean ± SD body mass index, kg/m^2^	26.1 ± 3.9
Mean ± SD body surface area, m^2^	1.8 ± 0.4

Native *T*_1_ values at 1.5T (*P* > 0.100) and 3.0T (*P* > 0.100) were normally distributed. The global mean native *T*_1_ relaxation time for all myocardial segments per subject was shorter at 1.5T (943.8 ± 24.7 msec) than at 3.0T (1154.7 ± 26.2 msec; *P* < 0.001). There was a moderate correlation between the intraindividual global native *T*_1_ values measured at different field strengths (*r* = 0.577; *P* < 0.001) (Supplementary Fig. 2).

No correlation was found between the LV ejection fraction and global native *T*_1_ relaxation times at 1.5T (*r* = 0.112; *P* = 0.343) or 3.0T (*r* = 0.204; *P* = 0.081).

### Artifact Analysis

Overall image quality was good (with 81.5% ranked as high, and 98.9% as high or adequate). After regional segmentation of the LV, 47 (3.5%) of 1344 segments imaged at 1.5T and 81 (6.0%) of 1344 segments at 3.0T were excluded because of artifacts related to susceptibility effects (76, 5.7%) and cardiorespiratory motion (52, 3.9%). The majority of excluded segments were located at the distal LV, especially at 3.0T (Supplementary Table 1). Motion artifacts were most common among older individuals and susceptibility artifacts were more common in males than in females (Supplementary Table 2).

### Regional *T*_1_ Values

We observed regional differences in mean native *T*_1_ relaxation times (Fig. 1, Supplementary Table 3). At 1.5T, mean native *T*_1_ values from septal segments (956.3 ± 44.4 msec) were longer than lateral segments (939.2 ± 54.2 msec; *P* < 0.001). The regional differences were similar at 3.0T for septal vs. lateral segments (1158.2 ± 45.9 vs. 1148.9 ± 56.9 msec; *P* = 0.012).

For the regional differences within the phantom *T*_1_ map, at 1.5T coefficients of variation were 0.4 for the smaller phantom and 0.3 for the larger phantom, and at 3.0T 0.8 and 0.4, respectively.

### Associations Between *T*_1_ With Gender and Age

**Figure 2:**
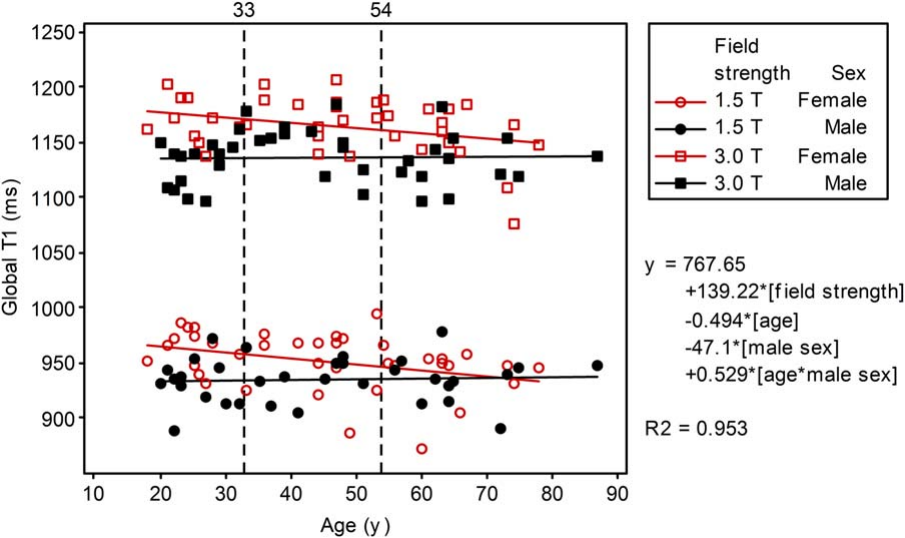
Global averaged myocardial native *T*_1_ relaxation times (mean, msec) displayed by age and sex at 1.5T and 3.0T.

The study population was categorized in tertiles of age (each *n* = 28): <33 years, 33–54 years, ≥55 years. In females, mean native *T*_1_ relaxation time reduced with increasing age (Fig. 2, Table 3). Native *T*_1_ did not vary with age in males. At 1.5T, global native *T*_1_ decreased by 5.50 msec for each additional decade (*P* = 0.014). Native *T*_1_ was shorter in men than in women (Table 3) with an interaction for global native *T*_1_ between age and sex (*P* = 0.046); ([mean global native *T*_1_ (ms)] = 976.0–0.550*[age]–44.8*[male sex]+0.619*[age*male sex]). Similar observations occurred at 3.0T (regression coefficient of –4.55 msec/decade [*P* = 0.042]). At 3.0T, global native *T*_1_ was shorter in males than in females (*P* = 0.001), but there was no interaction for age and sex (*P* = 0.143). Native *T*_1_ was multivariably independent of height, weight, and BSA at both field strengths (Table 4). Univariate relationship between native *T*_1_ and height, weight, and BSA was related to sex.

**Table 3:** Global Averaged Myocardial Native T1 Relaxation Times (Mean ± SD, msec) Grouped by Age Tertiles and Sex at 1.5T and 3.0T

	1.5T			3.0T		
	Males	Females	*P‐*values (males vs. females)	Males	Females	*P‐*values (males vs. females)
Age < 33, years (*n = * 28)	931.5 ± 22.2	960.0 ± 20.3	0.003	1130.2 ± 20.6	1166.5 ± 19.7	<0.001
Age 33‐54, years (*n = * 28)	936.8 ± 18.0	952.8 ± 28.5	0.093	1149.4 ± 23.7	1176.0 ± 20.6	0.006
Age ≥ 55, years (*n = * 28)	934.7 ± 22.3	937.7 ± 25.4	0.762	1132.3 ± 23.5	1153.0 ± 30.0	0.056
*P‐*value (tertiles)	0.828	0.092		0.079	0.045	

**Table 4:** Relationships Between Global Native T1 and Age, Sex, and Height, Weight, Body Mass Index, and Body Surface Area (*n = * 84)

Associations	1.5T		3.0T	
	Coefficient (95% CI)	*P‐*value	Coefficient (95% CI)	*P‐*value
Univariable				
Age, for 1 year difference	−0.228 (−0.556, 0.101)	0.171	−0.183 (−0.541, 0.174)	0.310
Male sex	−17.05 (−28.01, −6.08)	0.003	−28.55 (−39.51, −17.59)	<0.001
Height, for 10 cm difference	−5.78 (−11.59, 0.030)	0.051	−11.10 (−17.24, −4.96)	0.001
Weight, for 1 kg difference	−0.413 (−0.800, −0.026)	0.037	−0.430 (−0.848, −0.011)	0.044
BMI, for 1 kg/m^2^	−1.128 (−2.706, 0.451)	0.159	−0.189 (−1.846, 1.469)	0.821
BSA, for m^2^	−30.4 (−56.9, −3.9)	0.025	−40.6 (−69.2, −11.9)	0.006
Multivariable associations				
Age, for 1 year difference	−0.550 (−0.986, −0.115)	0.014	−0.455 (−0.893, −0.017)	0.042
Male sex	−44.8 (−74.0, −15.6)	0.003	−49.5 (−79.3, −19.8)	0.001
Age*male sex, interaction	0.619 (0.012, 1.225)	0.046	0.455 (−0.158, 1.068)	0.143
Height, for 10 cm difference	−1.63 (−9.08, 5.83)	0.664	−1.96 (−9.84, 5.92)	0.622
Weight, for 1 kg difference	−0.175 (−0.589, 0.238)	0.400	0.091 (−0.32, 0.502)	0.662
BSA, for m^2^	−12.4 (−42.8, 17.9)	0.415	2.1 (−29.1, 33.3)	0.894

Global native *T*_1_ was dependent on field strength (*P* < 0.001), and also on age (*P* = 0.002), sex (*P* < 0.001), and an interaction for native *T*_1_ between age and sex included in the regression equation (*P* = 0.016); ([mean global native *T*_1_ (msec)] = 767.65 + 139.22*[field strength]‐0.494*[age]‐47.1*[male sex]+0.529*[age*male sex]).

### Myocardial Extracellular Volume in Volunteers Aged >45 Years

In all, 37 (88.1%) of 42 volunteers aged >45 years underwent contrast‐enhanced MRI. None of them had evidence of myocardial fibrosis (scar), based on the late gadolinium enhancement imaging. HCT was available for 26 (70.3%) volunteers aged >45 years, and an estimated HTC value used for 11 (29.7%) volunteers. There was no difference between actual (0.415 ± 0.026) and estimated (0.408 ± 0.034; *P* = 0.557) HCT values. ECV measurements were available for 514 of 592 (86.8%) segments. ECV values were normally distributed (*P* = 0.086). The mean ECV fraction was similar in septal (25.3 ± 3.1%) and lateral segments (25.5 ± 2.9%; *P* = 0.776). The mean global ECV fraction (25.0 ± 2.3%) was not associated with sex (*P* = 0.071), age (*P* = 0.147), or body size (BMI: *P* = 0.760, BSA: *P* = 0.583), with correlation for ECV fraction and age (*r* = –0.213; *P* = 0.205) remaining weak when grouped by sex (ECV% + male age: *r* = –0.248; *P* = 0.322, and ECV% + female age: *r* = –0.165; *P* = 0.499).

### Intra‐ and Interobserver Agreement of *T*_1_ Measurements

At 1.5T the intraclass correlation coefficient for reliability of mean *T*_1_ was 0.913 (95% confidence interval [CI]: 0.790, 0.960; *P* < 0.001), and at 3.0T 0.909 (95% CI: 0.808, 0.958; *P* < 0.001). Bland–Altman plots showed no evidence of bias. The intraobserver coefficients of variation for mean *T*_1_ were 2.07 (1.5T) and 2.21 (3.0T), and for interobservers 2.79 (1.5T) and 2.83 (3.0T). The intra‐ and interobserver coefficients of variation were slightly greater for lateral (vs. septal) regions at both field strengths (Supplementary Table 4).

## Discussion

We present information on myocardial native *T*_1_ values at 1.5T and 3.0T in 84 adults across a broad age range. The mean age in our study was older than in the largest other study of native *T*_1_ to date.^9^ We used contrast‐enhanced MRI to rule out the possibility of incidental myocardial disease in older subjects.

Native *T*_1_ was shorter at 1.5T vs. 3.0T, as would be expected. Whereas *T*_2_ relaxation times remain fairly constant, *T*_1_ relaxation times have been shown to be longer for most tissues at higher field strengths.^20^ Our measurements of myocardial *T*_1_ values at different field strengths are consistent with those from other water‐based tissues elsewhere in the body.^20^ Our main observation was an age‐related decline in global mean native *T*_1_ values in females. Myocardial native *T*_1_ relaxation times were longer in young females than in young males. Native *T*_1_ relaxation time was not associated with age in males. Second, native *T*_1_ relaxation times were longer in the LV septum vs. lateral wall. These differences were mostly consistent across both MRI field strengths. Third, we observed that the age‐ and sex‐related associations were independent of field strength and body size. Fourth, as would be expected, we found that cardiorespiratory motion artifacts and susceptibility effects were more common at the higher field (3.0T vs. 1.5T) and predominated in the distal regions of the LV. A higher incidence of motion artifacts occurred in older patients (≥55 years), and susceptibility artifacts were more common among males. Finally, the ECV fraction was not associated with myocardial region, age, or sex, in contrast to native *T*_1_ values.

Previous studies have reported conflicting information on age or sex associations of myocardial native *T*_1_ relaxation times.^9^, ^21^, ^22^, ^23^ Most of these investigations focused on comparisons between age groups, while we present regression analysis data independent of grouping and include an interaction term for age and male sex. Although we do not present paired longitudinal data for native *T*_1_ measurements over time in the same individuals, the data suggest an age‐related decline in myocardial native *T*_1_ in females, in contrast to some previous studies.^21^, ^22^ These studies have interpreted an age‐related elevation in myocardial native *T*_1_ values as a sign of increased myocardial fibrosis.^24^, ^25^ However, ECV values observed in our sample were not consistent with age‐related myocardial fibrosis among older subjects, even though our sample included elderly subjects. Sex differences in age‐related changes are especially relevant, as females develop cardiovascular disease on average 7–10 years later than men.^26^, ^27^ Our observations raise the question of whether sex hormone status may influence myocardial tissue characteristics as reflected by myocardial native *T*_1_. Estrogen, progesterone, and androgens have effects on myocardial structure and function.^28^, ^29^ For example, sex‐specific differences in myocardial hypertrophy have been recently associated with the regulatory role of estrogen pathways.^30^ We present our results and interpretation as hypothesis generating and further research is warranted.

Our observation of regional differences in native myocardial *T*_1_ relaxation times are in line with previously reported findings.^21^, ^31^ Although B1 inhomogeneities may have a small effect, based on our phantom assessment they are unlikely to be the cause of regional differences observed in the myocardial *T*_1_ values. Instead, the lateral free wall is known to be more prone to motion artifacts,^32^ which may partly explain the lower native *T*_1_ values in the lateral segments. As *T*_1_ maps are derived from a sequential series of images, motion during the acquisition may result in a poor *T*_1_ model fit and, consequently, in falsely low *T*_1_ values.^1^ This seems to be further supported by our finding of a higher variation of *T*_1_ values in lateral (vs. septal) regions. The smaller spread of septal values (vs. lateral) supports the septal sampling approach proposed by Rogers et al for the standardization of native *T*_1_ measurements.^31^

Limited data are available about the reproducibility of myocardial *T*_1_ relaxation times at different field strengths. The sources of variation between myocardial *T*_1_ at different field strengths are likely to involve measurement errors in acquisition and analyses. Partial volume effects that are a recognized technical limitation^9^ are likely to be more relevant in the distal LV and lateral wall where motion is greatest. MRI artifacts are more common at the higher magnetic field and affect especially distal LV.^33^, ^34^ Increased incidence of motion artifacts among older subjects may reflect a reduced propensity for breath‐holding during the MRI acquisition,^32^ whereas the higher incidences of susceptibility artifacts among males may be related to the sex differences in LV dimensions.^35^ Consistent with our findings, motion and susceptibility artifacts have been shown to be more prevalent at higher field strengths also in other body parts, such as the boundaries of para‐nasal sinuses and bone–soft tissue interfaces in the spinal canal.^36^ These artifacts are often subtle, calling for caution in clinical use.

The *T*_1_ mapping field is progressing rapidly with emerging clinical utility. The first *T*_1_ consensus statement of the Society for Cardiovascular Magnetic Resonance and CMR Working Group of the European Society of Cardiology^8^ highlights the importance of representative local normal values for each site/scanner. Our data have a slightly lower spread of global and local mean *T*_1_ values than what has been reported in the myocardial reference ranges obtained before.^37^, ^38^, ^39^ It is expected that in the future advances in MRI hardware and postprocessing will lower the spread even further.^20^ A major limitation of our study is that we did not collect information on reproductive history, menopause, or sex hormone status. Considering the known precision of *T*_1_ measurements,^39^ our finding of a relatively low correlation between *T*_1_ values at 1.5T and 3.0T raises some important questions. The lower than expected interscan reproducibility of *T*_1_ values may be partly related to the higher flip angle (35°) at 3.0T. A high flip angle may bias the *T*_1_ estimations with MOLLI sequences, especially at higher field strengths, and the use of smaller flip angles than what was applied in our study has been recently proposed (recommended: 1.5T: 30°, and 3.0T: 20°).^35^ Further limitations include large but limited numbers of volunteers and the delay between the scans at different field strengths. Finally, MOLLI sequences are known to systematically underestimate true *T*_1_ values, since the later images are influenced by the previous inversions. Relying on R‐R intervals for the timings^12^ results in *T*_1_ estimations being easily affected by incomplete tissue recovery between inversions, especially at higher heart rates. Due to the effects of incomplete recovery, it has been suggested that different MOLLI schemes should be employed for native and postcontrast *T*_1_ measurements.^40^

In conclusion, native *T*_1_ values vary according to myocardial location. The explanation may be related to myocardial structure/function relationships as well as regional variation in artifacts. Sex difference in global myocardial mean native *T*_1_ relaxation times are observed among younger but not older subjects and this observation was consistent between MRI field strengths.

## Funding

This research was supported by a Project Grants from the Chief Scientist Office (SC01), Medical Research Scotland (343 FRG) and the British Heart Foundation (BHF‐PG/14/64/31043). Dr Mangion is supported by a Fellowship from the British Heart Foundation (FS/15/54/31639).

## Supporting information

Additional supporting information may be found in the online version of this article.

Supporting Information

## References

[1] Ferreira V , Piechnik S , Dall'Armellina E , et al. Non‐contrast T1‐mapping detects acute myocardial edema with high diagnostic accuracy: a comparison to T2‐weighted cardiovascular magnetic resonance. J Cardiovasc Magn Reson 2012;14:42. 22720998 10.1186/1532-429X-14-42 PMC3424120

[2] Ferreira V , Piechnik S , Dall'Armellina E , et al. Native T1‐mapping detects the location, extent and patterns of acute myocarditis without the need for gadolinium contrast agents. J Cardiovasc Magn Reson 2014;16:36. 24886708 10.1186/1532-429X-16-36 PMC4041901

[3] Karamitsos T , Piechnik S , Banypersad S , et al. Noncontrast T1 mapping for the diagnosis of cardiac amyloidosis. JACC Cardiovasc Imaging 2013;6:488–497. 23498672 10.1016/j.jcmg.2012.11.013

[4] Sado D , White S , Piechnik S , et al. Identification and assessment of Anderson‐Fabry disease by cardiovascular magnetic resonance noncontrast myocardial T1 mapping. Circ Cardiovasc Imaging 2013;6:392–398. 23564562 10.1161/CIRCIMAGING.112.000070

[5] Flett A , Sado D , Quarta G , et al. Diffuse myocardial fibrosis in severe aortic stenosis: an equilibrium contrast cardiovascular magnetic resonance study. Eur Heart J Cardiovasc Imaging 2012;13:819–826. 22634740 10.1093/ehjci/jes102

[6] Scholz T , Martins J , Skorton D . NMR relaxation times in acute myocardial infarction: relative influence of changes in tissue water and fat content. Magn Reson Med 1992;23:89–95. 1734185 10.1002/mrm.1910230110

[7] Oshinski J , Delfino J , Sharma P , Gharib A , Pettigrew R . Cardiovascular magnetic resonance at 3.0T: current state of the art. J Cardiovasc Magn Reson 2010;12:55. 20929538 10.1186/1532-429X-12-55 PMC2964699

[8] Moon J , Messroghli D , Kellman P , et al. Myocardial T1 mapping and extracellular volume quantification: a Society for Cardiovascular Magnetic Resonance (SCMR) and CMR Working Group of the European Society of Cardiology consensus statement. J Cardiovasc Magn Reson 2013;15:92. 24124732 10.1186/1532-429X-15-92 PMC3854458

[9] Piechnik S , Ferreira V , Lewandowski A , et al. Normal variation of magnetic resonance T1 relaxation times in the human population at 1.5 T using ShMOLLI. J Cardiovasc Magn Reson 2013;15:13. 23331520 10.1186/1532-429X-15-13 PMC3610210

[10] Messroghli D , Radjenovic A , Kozerke S , Higgins D , Sivananthan M , Ridgway J . Modified Look‐Locker inversion recovery (MOLLI) for high‐resolution T1 mapping of the heart. Magn Reson Med 2004;52:141–146. 15236377 10.1002/mrm.20110

[11] Xue H , Guehring J , Srinivasan L , et al. Evaluation of rigid and non‐rigid motion compensation of cardiac perfusion MRI. Med Image Comput Comput Assist Intervent 2008, Pt Ii, Proc 2008;5242:35–43. 10.1007/978-3-540-85990-1_5 18982587

[12] Messroghli D , Greiser A , Frohlich M , Dietz R , Schulz‐Menger J . Optimization and validation of a fully‐integrated pulse sequence for modified Look–Locker inversion‐recovery (MOLLI) T1 mapping of the heart. J Magn Reson Imaging 2007;26:1081–1086. 17896383 10.1002/jmri.21119

[13] Kellman P , Arai A , McVeigh E , Aletras A . Phase‐sensitive inversion recovery for detecting myocardial infarction using gadolinium‐delayed hyperenhancement. Magn Reson Med 2002;47:372–383. 11810682 10.1002/mrm.10051 PMC2041905

[14] Chuang M , Gona P , Hautvast G , et al. CMR reference values for left ventricular volumes, mass, and ejection fraction using computer‐aided analysis: the Framingham Heart Study. J Magn Reson Imaging 2014;39:895–900. 24123369 10.1002/jmri.24239 PMC3961548

[15] Kramer C , Barkhausen J , Flamm S , Kim R , Nagel E . Standardized cardiovascular magnetic resonance imaging (CMR) protocols, society for cardiovascular magnetic resonance: board of trustees task force on standardized protocols. J Cardiovasc Magn Reson 2008;10:35. 18605997 10.1186/1532-429X-10-35 PMC2467420

[16] Cerqueira M , Weissman N , Dilsizian V , et al. Standardized myocardial segmentation and nomenclature for tomographic imaging of the heart: a statement for healthcare professionals from the Cardiac Imaging Committee of the Council on Clinical Cardiology of the American Heart Association. Circulation 2002;105:539–542. 11815441 10.1161/hc0402.102975

[17] de Ravenstein C , Bouzin C , Lazam S , et al. Histological Validation of measurement of diffuse interstitial myocardial fibrosis by myocardial extravascular volume fraction from modified Look‐Locker imaging (MOLLI) T1 mapping at 3 T. J Cardiovasc Magn Reson 2015;17. 26062931 10.1186/s12968-015-0150-0 PMC4464705

[18] Treibel T , Fontana M , Maestrini V , et al. Synthetic ECV: simplifying ECV quantification by deriving haematocrit from T1 blood. Heart 2015;101:A16–A17.

[19] Du Bois D , Du Bois E . A formula to estimate the approximate surface area if height and weight be known. Arch Intern Med 1916;17:863–871. 2520314

[20] Gold GE , Han E , Stainsby J , et al. Musculoskeletal MRI at 3.0 T: relaxation times and image contrast. Am J Roentgenol 2004;183;343–351. 15269023 10.2214/ajr.183.2.1830343

[21] Dabir D , Child N , Kalra A , et al. Reference values for healthy human myocardium using a T1 mapping methodology: results from the International T1 Multicenter cardiovascular magnetic resonance study. J Cardiovasc Magn Reson 2014;16:69. 25384607 10.1186/s12968-014-0069-x PMC4203908

[22] Liu C , Liu Y , Wu C , et al. Evaluation of age‐related interstitial myocardial fibrosis with cardiac magnetic resonance contrast‐enhanced T1 mapping: MESA (Multi‐Ethnic Study of Atherosclerosis). J Am Coll Cardiol 2013;62:1280–1287. 23871886 10.1016/j.jacc.2013.05.078 PMC3807823

[23] Piechnik S , Ferreira V , Lewandowski A , et al. Age and gender dependence of pre‐contrast T1‐relaxation times in normal human myocardium at 1.5T using ShMOLLI. J Cardiovasc Magn Reson 2012;14:P221.

[24] Kellman P , Wilson J , Xue H , Ugander M , Arai A . Extracellular volume fraction mapping in the myocardium, part 1: evaluation of an automated method. J Cardiovasc Magn Reson 2012;14. 22963517 10.1186/1532-429X-14-63 PMC3441905

[25] Florian A , Ludwig A , Rosch S , Yildiz H , Sechtem U , Yilmaz A . Myocardial fibrosis imaging based on T1‐mapping and extracellular volume fraction (ECV) measurement in muscular dystrophy patients: diagnostic value compared with conventional late gadolinium enhancement (LGE) imaging. Eur Heart J Cardiovasc Imaging 2014;15:1004–1012. 24686257 10.1093/ehjci/jeu050

[26] Kannel W , Wilson P . Risk‐factors that attenuate the female coronary disease advantage. Arch Intern Med 1995;155:57–61. 7802521

[27] Rosano G , Vitale C , Marazzi G , Volterrani M . Menopause and cardiovascular disease: the evidence. Climacteric 2007;10:19–24. 17364594 10.1080/13697130601114917

[28] Mendelsohn M , Karas R . Molecular and cellular basis of cardiovascular gender differences. Science 2005;308:1583–1587. 15947175 10.1126/science.1112062

[29] Regitz‐Zagrosek V . Therapeutic implications of the gender‐specific aspects of cardiovascular disease. Nat Rev Drug Discov 2006;5:425–438. 16672926 10.1038/nrd2032

[30] van Eickels M , Grohe C , Cleutjens J , Janssen B , Wellens H , Doevendans P . 17 beta‐estradiol attenuates the development of pressure‐overload hypertrophy. Circulation 2001;104:1419–1423. 11560859 10.1161/hc3601.095577

[31] Rogers T , Dabir D , Mahmoud I , et al. Standardization of T1 measurements with MOLLI in differentiation between health and disease: the ConSept study. J Cardiovasc Magn Reson 2013;15:78. 24025486 10.1186/1532-429X-15-78 PMC3847466

[32] Ferreira P , Gatehouse P , Mohiaddin R , Firmin D . Cardiovascular magnetic resonance artefacts. J Cardiovasc Magn Reson 2013;15:14. 23697969 10.1186/1532-429X-15-41 PMC3674921

[33] Kellman P , Hansen M . T1‐mapping in the heart: accuracy and precision. J Cardiovasc Magn Reson 2014;16:2. 24387626 10.1186/1532-429X-16-2 PMC3927683

[34] Wieben O , Francois C , Reeder S . Cardiac MRI of ischemic heart disease at 3 T: potential and challenges. Eur J Radiol 2008;65:15–28. 18077119 10.1016/j.ejrad.2007.10.022

[35] Sado D , Flett A , Banypersad S , et al. Cardiovascular magnetic resonance measurement of myocardial extracellular volume in health and disease. Heart 2012;98:1436–1441. 22936681 10.1136/heartjnl-2012-302346

[36] Farahani K , Sinha U , Sinha S , Chiu LC , Lufkin RB . Effect of field strength on susceptibility artifacts in magnetic resonance imaging. Comput Med Imaging Graph 1990;14:409–413. 2272012 10.1016/0895-6111(90)90040-i

[37] von Knobelsdorff‐Brenkenhoff F , Prothmann M , Dieringer M , et al. Myocardial T1 and T2 mapping at 3 T: reference values, influencing factors and implications. J Cardiovasc Magn Reson 2013;15:53. 23777327 10.1186/1532-429X-15-53 PMC3702448

[38] Nacif M , Turkbey E , Gai N , et al. Myocardial T1 mapping with MRI: comparison of Look‐Locker and MOLLI sequences. J Magn Reson Imaging 2011;34:1367–1373. 21954119 10.1002/jmri.22753 PMC3221792

[39] Kawel N , Nacif M , Zavodni A , et al. T1 mapping of the myocardium: intra‐individual assessment of post‐contrast T1 time evolution and extracellular volume fraction at 3T for Gd‐DTPA and Gd‐BOPTA. J Cardiovasc Magn Reson 2012;14:26. 22540153 10.1186/1532-429X-14-26 PMC3405486

[40] McDiarmid AK , Broadbent DA , Higgins DM , et al. The effect of changes to MOLLI scheme on T1 mapping and extra cellular volume calculation in healthy volunteers with 3 Tesla cardiovascular magnetic resonance imaging. Quant Imaging Med Surg 2015;5:503–510. 26435913 10.3978/j.issn.2223-4292.2015.04.07 PMC4559984

