# Supplementary material for: Native myocardial longitudinal (T 1) relaxation time: Regional, age, and sex associations in the healthy adult heart
Source: J Magn Reson Imaging. 2016 Mar 4;44(3):541–8. doi: 10.1002/jmri.25217 (PMC5025725; doi:10.1002/jmri.25217)
Supplement: Supplementary file 1 — Supporting Information [file JMRI-44-541-s001.docx]

## Supplementary material

Table 1 Artefact analysis, grouped by slice and segment, at 1.5 T and 3.0 T

| Number of segments excluded due to artefact, n (%) | 1.5 T | 3.0 T |
| --- | --- | --- |
| Grouped by slice  Basal  Mid-ventricular  Apical | 12 (2.7%)  18 (4.0%)  17 (5.7%) | 18 (3.8%)  21 (4.4%)  42 (13.1%) |
| Grouped by segment  Anterior  Anterolateral  Inferolateral  Lateral  Inferior  Inferoseptal  Anteroseptal  Septal | 13 (5.8%)  3 (2.0%)  6 (4.0%)  4 (5.3%)  9 (4.0%)  5 (3.3%)  4 (2.7%)  3 (4.0%) | 20 (8.3%)  11 (6.9%)  10 (6.3%)  21 (26.3%)  10 (4.2%)  3 (1.9%)  2 (1.3%)  4 (5.0%) |

Table 2 Distribution of artefacts, grouped by age, sex and type

| Number of segments excluded due to artefact, n (%) | All artefacts | Susceptibility artefacts | Motion artefacts |
| --- | --- | --- | --- |
| Grouped by age  Age <33, years (n=28)  Age 33-54, years (n=28)  Age ≥55, years (n=28) | 31 (1.3%)  44 (1.9%)  53 (2.3%) | 25 (1.1%)  25 (1.1%)  26 (1.1%) | 6 (0.3%)  19 (0.8%)  27 (1.1%) |
| Grouped by sex  Males (n=43)  Females (n=41) | 88 (3.7%)  40 (1.7%) | 58 (2.5%)  18 (0.8%) | 30 (1.3%)  22 (0.9%) |

**Table 3** Segmental myocardial native T1 values (ms)

|  | 1.5 T | | 3.0 T | |
| --- | --- | --- | --- | --- |
|  | Male | Female | Male | Female |
| Basal  Anterior  Anterolateral  Inferolateral  Inferior  Inferoseptal  Anteroseptal | 939.1±30.8  927.4±43.1  954.8±32.2  950.0±35.3  947.6±28.3  940.3±29.5 | 943.4±43.9  936.1±44.1  981.8±41.6  973.3±35.3  976.6±28.4  972.0±33.7 | 1113.0±37.0  1118.9±38.0  1127.1±43.0  1138.5±44.3  1135.0±26.1  1136.5±29.8 | 1145.0±43.6  1143.0±43.1  1148.8±40.6  1159.0±33.7  1163.6±30.0  1161.5±32.7 |
| Mid-ventricular  Anterior  Anterolateral  Inferolateral  Inferior  Inferoseptal  Anteroseptal | 918.4±32.1  915.6±41.6  938.5±35.1  935.7±31.7  944.0±41.5  932.8±32.2 | 921.6±43.5  909.9±34.2  947.8±38.1  948.3±38.4  965.5±30.8  955.2±43.8 | 1127.8±42.3  1136.8±40.2  1127.0±45.4  1123.3±38.4  1145.6±28.5  1145.6±31.1 | 1141.7±40.7  1162.1±40.1  1156.9±42.1  1155.1±44.2  1161.0±43.1  1161.2±44.7 |
| Apical  Anterior  Lateral  Inferior  Septal | 930.1±38.4  930.1±41.4  948.7±38.7  951.4±31.2 | 935.1±41.6  936.4±34.1  958.0±43.0  974.4±46.9 | 1152.9±42.7  1162.6±46.4  1118.9±46.5  1150.6±42.2 | 1195.1±40.5  1220.9±38.9  1186.8±41.3  1214.7±40.6 |

**Table 4** Coefficients of variation for T1 values, per ventricular wall territory

|  | 1.5 T | 3.0 T |
| --- | --- | --- |
| Septal ROIs  Intra-observer (n=30)  Inter-observer (n=30)  Lateral ROIs  Intra-observer (n=30)  Inter-observer (n=30) | 1.91  1.97  2.23  2.41 | 2.64  2.70  2.97  3.08 |

### Supplementary Figure Legends

**Figure 1** Mid-ventricular T1 maps at 1.5 T and 3.0 T: a) in a young (32 years) and b) an older (67 years) female subject. Gadolinium-based contrast was not given to adults<45 years of age

**Figure 2** Within-subject global native T1 values measured at different field strengths (1.5 T and 3.0 T)


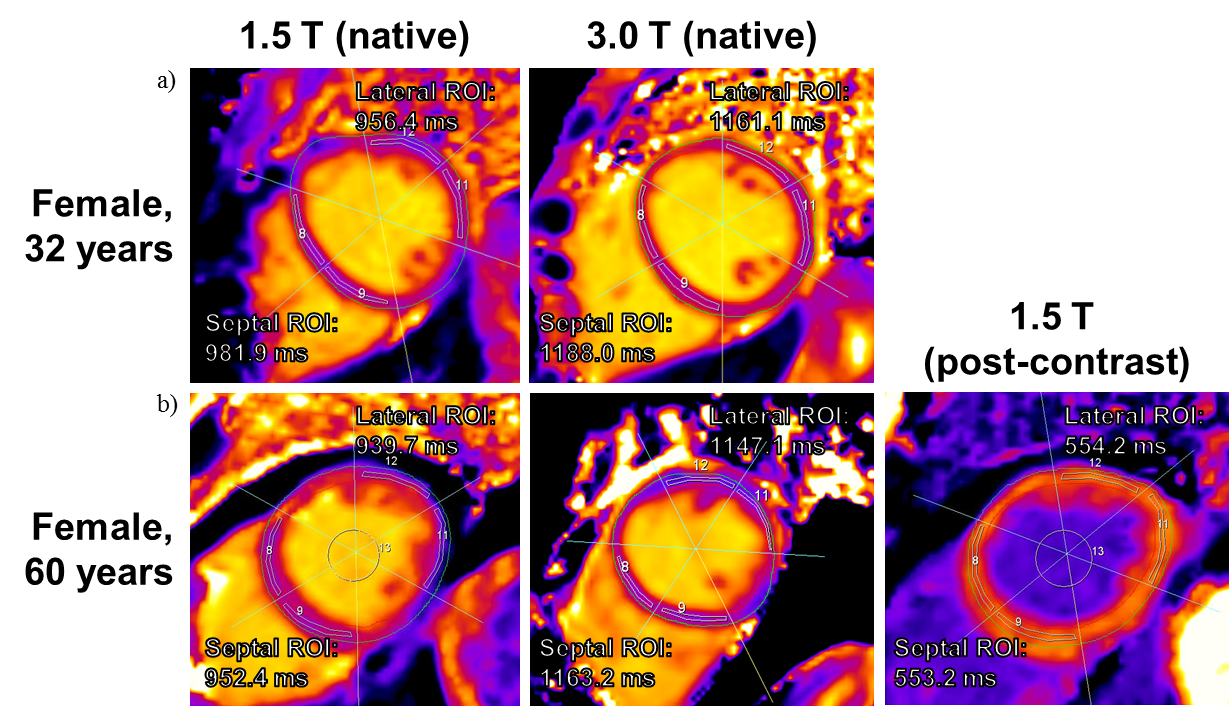


**Figure 1** Mid-ventricular T1 maps at 1.5 T and 3.0 T: a) in a young (32 years) and b) an older (67 years) female subject. Gadolinium-based contrast was not given to adults<45 years of age

**Figure 2** Intra-individual global native T1 values measured at different field strengths (1.5 T and 3.0 T)
